# Assessment of aflatoxin in milk and feed samples and impact of seasonal variations in the Punjab, Pakistan

**DOI:** 10.1002/fsn3.1557

**Published:** 2020-04-14

**Authors:** Naveed Akbar, Muhammad Nasir, Naureen Naeem, Mansur‐ud‐Din Ahmad, Farhan Saeed, Faqir Muhammad Anjum, Sanaullah Iqbal, Muhammad Imran, Tabussam Tufail, Faiz‐ul‐Hassan Shah, Muhammad Atif

**Affiliations:** ^1^ Department of Food Science and Human Nutrition University of Veterinary and Animal Sciences Lahore Pakistan; ^2^ Department of Epidemiology and Public Health University of Veterinary and Animal Sciences Lahore Pakistan; ^3^ Institute of Home and Food Sciences Government College University Faisalabad Pakistan; ^4^ University of The Gambia Gambia Gambia; ^5^ University Institute of Diet and Nutritional Sciences Faculty of Allied Health Sciences The University of Lahore Lahore Pakistan; ^6^ Department of Clinical Laboratory Sciences College of Applied Medical Sciences Jouf University Kingdom of Saudi Arabia Sakaka

**Keywords:** aflatoxin B1, aflatoxin M1, feed ingredients, mycotoxins

## Abstract

The present study was designed to assess the incidence of aflatoxin contamination in animal feed and raw milk samples (total 240 each) collected from dairy farms during the complete year of 2015. These samples were collected through a cluster random sampling technique by dividing the province of Punjab, Pakistan into five clusters (north, south, east, west and central). Factors (environmental & physical) affecting aflatoxin contamination in milk and animal feed at farms were also studied. The AFM1 levels in raw milk & AFB1 levels in feed samples were analyzed by using the ELISA technique. Results demonstrated that overall about 53% raw milk samples from dairy farms were contaminated beyond the US MRL (0.50 µg/L) for AFM1 with than average level of 0.59 µg/L, while the 95% farm feed samples were exceeding the FDA MRL (20 µg/kg) of AFB1 with average level of 43 µg/kg. During winter season, the concentration of AFM1 was higher in all clusters with avg 0.68 µg/L, while the AFB1 contamination was highest in the spring season with avg 54 µg/kg. Market feed prices had negative correlation with AFB1 contamilevels, which were further supported by the positive correlation between quantity of feed at farms with AFM1 and AFB1 contamination. Results exhibited significantly positive impact of environmental factors on milk and feed aflatoxin contamination levels, whereas temperature showed an inverse relationship with AFM1 and AFB1 levels. The study recommends need of synergistic extension work to support dairy farms and highlight the contamination levels for regulatory bodies to introduce strategic policies for control measures.

## Graphical abstract

Study recommends dire need of synergistic extension work for the awareness and motivation of farmers. Moreover, this extensive screening facility highlights government authorities to establish mechanism of support required to the farmers in postharvest handling, drying, storage, and transportation of agriculture produce. Strategic actions and policies must implement to make aflatoxin‐free milk supply chain.

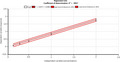

## 1 INTRODUCTION

Mycotoxins are the group of naturally prevailing secondary metabolites, mainly produced by filamentous fungi (Varga, Frisvad, & Samson, 2011). This is the global problem and a quarter of the world's food and food products are affected (Lawlor & Lynch, 2005). There are many types of mycotoxins among which AFB1 is the most abundant and toxic due to teratogenic and mutagenic effects (Kang'ethe & Lang'a, 2009). The chief source of aflatoxins are *Aspergillus flavus, A. parasiticus* and *A. nomius* (Mostrom & Jacobsen, 2011).

AFM1 is documented as a metabolite of AFB1 and is concealed in the milk of those animals’ which are fed on contaminated feed (Ruangwises & Ruangwises, 2010). It starts appearing in milk after 12–24 hr of contaminated feed ingestion (Rahimi et al., 2012). It must be remembered that conversion factor of AFB1 from animal feed to AFM1 in raw milk is 0.30%–6.2%, depending upon the genetics, lactation stage, milk production and heathe animalsdition of animals (Unusan, 2006). Milk and its products are imperative for sustained human health (Bașkaya, Aydın, Yıldız, & Bostan, 2006). Aflatoxin toxicity thought to be the one of the major causes of liver cancer (Omata et al., 2010). Hepatocellular carcinoma (HCC) risk reported to increase 30 folds in the presence of aflatoxin and hepatitis B virus. Initially the International Agency for Research on Cancer (IARC) based on the toxicity, classified AFM1 as agent in Group 2B possessing potentially carcinogenic influence on human health while further it was reclassified to Group 1, along with AFB1 carcinogenic agent (IARC, 2002). Furthermore, thermal processing like pasteurization and even ultra‐high temperature (UHT) treatments are unproductive in abolishing or reducing harm of contamination because of its stability at high temperatures (Prandini et al., 2009).

Livestock sector is inevitable for the economic development of any country. Pakistan is blessed with variety of animals and graced with third position among world largest milk producing nations with annual production of 54 million tons (Iqbal, Iqbal, Akbar, Khan, & Abbas, 2015). It contributes about 46.8% to agriculture with 10%–25% income generated by rural people through livestock (Iqbal, Ahmad, & Jehangir, 1999). Livestock plays vital role in alleviating poverty of rural areas by providing food and income (Mahmood, Khalid, & Kouser, 2009). Animals fed on these contaminated sources were observed with a decrease in growth rate, milk production, milk quality attributes and ultimately with compromised desired immunity against infections (Akande, Abubakar, Adegbola, & Bogoro, 2006). Aflatoxicosis in cattle leads to lethargy, ataxia, rough hair coat, enlarged pale fatty liver, less feed intake by loss of appetite, diarrhea, blindness, teeth grinding, frothing at the mouth, abortion, lameness, ovarian cyst and other reproductive disorders (Nibbelink, 1986; Pirestani, Tabatabaei, Fazeli, Antikchi, & Baabaei, 2011).

In order to minimize the risk of contamination in feed and milk, regulatory restrictions have been imposed across the world. In China, the limit of AFB1 in feed is 10 µg/kg while limits for milk is 0.5 µg/L (Wang & Liu, 2008). Most of the other countries including US Food and Drug Administration (FDA) established 0.5 µg/L MRL (maximum residual limit) for milk. European Commission Directive of 2004 described that 0.05 and 0.025 µg/L limits for liquid and dried milk, respectively (Commission, Programme, & Organization, 2007). Pakistan's province of Punjab has also set the levels of AFM1 in raw milk for processing as 5 and 0.5 µg/L milk for consumption under the legal frame work of Punjab Food Authority Regulations 2018 (PFA, 2018). Pakistan has also set the legal limits of Animal feedstuffs in the Punjab Feed Stuff and Compound Feed Act, 2016. According to that act the compound feed or concentrate manufactured for lactating dairy cow should not contain AFB1 above 50 µg/kg, while for other commonly used feed ingredients, AFB1 levels are different in Act as presented in Table 1.

**Table 1:** Acceptable AFB1 levels of commonly used feed stuffs, according to the Punjab Feed Stuff and Compound Feed Act, 2016

Ingredients/feedstuffs	Max. AFB1 (µg/kg)
Cotton seed cake (*Khal Banola*)	200
Cotton seed	200
Cotton seed meal	200
Rice polish	50
Mazie or corn gluten	100
Maize or corn gluten meal	100
Naan or Roti Tukra	200
Confectionaries waste or by products	200

Pakistan has been facing huge economic losses so far due to the lack of aflatoxin action plan and its implementation. Investigation report of PCSIR, Karachi shared a report during 2017, when approximately five‐hundred dairy animal died and twelve hundred fell sick due to the feeding of highly contaminated mycotoxin feed (Sultana & Hanif, 2009). Similarly, few years ago many of Pakistani export consignments were rejected and banned owing to high aflatoxin contamination like custard powder from South Korea, peanut from UK and chili powder from Europe.

Keeping in view the above details, the present study are designed (a) to highlight the presence of AFM1 & AFB1 contamination in raw milk and animal feed samples from different farms of province of Punjab, Pakistan (b) to investigate the environmental impact (temperature, season, humidity) on AFM1 & AFB1 levels in milk and feed samples (c) to compare the samples which exceeds the permissible level of location and international regulations and (d) to construct a database that could help policy maker in local governments, law enforcement agencies, dairy farmers and consumers to focus immediate attention to prevent or minimize the health risks associated from these toxins.

## 2 MATERIAL AND METHODS

### 2.1 Sampling plan

Punjab province of Pakistan consist of thirty six districts, which were divided into five clusters as north, south, east, west & central. The division plan of clusters for sampling is described in our previous publication (Akbar et al., 2019). Farmer having milk yield more than forty liters per day was selected from tehsils (an administrative unit of district) by using simple random sampling. In Punjab province corn, concentrate feed mix, bread pieces, cotton seed cake and rice polish are the most common animal feed sources. So, keeping in view the usage of feed in a sampling month, high consuming feed of that month is selected for AFB1 analysis.

### 2.2 Samples collection

In this study, milk & feed samples, 240 each were collected from dairy farms situated in each cluster (North, South, East, West and Central) of Punjab Pakistan during the year 2015. Four milk and feed samples were collected at different times within a month up to a year. The plastic bottles having volume capacity of 200 ml filled with milk samples and these bottles were place in a sampling cooler with icepacks and crushed ice during transportation. The milk samples were either analyzed immediately or stored in freezer in case of delayed analysis. Feed samples were transported with in sampling cooler with ice pads and in lab were placed in refrigerator at 2–8°C to avoid any fungal growth acceleration in a packed feed until analyzed.

### 2.3 Sample preparation & method validation for AFM1 analysis

The competitive enzyme immunoassay using Elisa kit Ridascreen^®^ Fast Aflatoxin M1, R5812 (R‐Biopharm AG) was used for quantitative analysis of AFM1 in the milk samples. Sample preparation was done by following the manual instructions, while method validation was performed as details are published in our previous study (Akbar et al., 2019).

### 2.4 Sample preparation for AFB1 analysis

Veratox^®^, (product # 8030) manufactured by Neogen, is a direct competitive ELISA using polyclonal antibodies and is approved by Grain Inspection, Packers and Stockyards Administration (GIPSA) and FGIS. Each sample (250 g) was thoroughly mixed and ground to a fine powder by Grindomix knife mill (GM 200, Retsch) and later grinded material passes through a 20‐mesh sieve. Aflatoxin was extracted by weighing 25.0 g ± 0.1 g of finely ground test portion out of 250g and mixed into 250 ml media solution bottle (Pyrex 1395–250) having measure quantity of 125 ml methanol and water (70 + 30) solution. Homogenized for 2 min ± 20 s at high speed using a homogenizer (e.g., Ultra‐Turrax T18). Sample allowed to settled for 2 min to 3 min after extracting to enable some of the sample to settled before filtering the extract. Filtered the extract by pouring at least 5 ml through Whatman filter paper no 1 and stored the filtrate into a 125‐ml amber glass Erlenmeyer.

### 2.5 Procedure and validation for AFB1 analysis by ELISA

Manufacturer's recommendations were followed throughout the analysis. Blue‐labelled bottles having conjugate were pipetted 100 μl in each red‐marked mixing well. Standards (0.00, 5.00, 15.00 and 50.00 μg/kg) were added 100 μl by using a new pipette tip for each one was transferred into the wells. Liquids were mixed by using a 12‐channel pipettor by pipetting it up and down five times before transferring 100 μl of each red marked mixing well to the antibody coated wells. Each well was incubated for 2 min at room temperature (20–25°C) and then drain the liquid contents of the antibody wells into a waste container. Distilled water (at least 250 μl) added to wash the wells by using 12‐channel pipette and dumped them out. Washing step repeated five times. Wells were turned upside‐down and tap out on a paper towel until the remaining water has been removed. With new tips on the 12‐channel, 100 μl of substrate were added into the each well and mixed them gently by sliding microwell holder back and forth on a flat surface for 10–20 s without splashing reagents from the wells before incubation for 3 min at room temperature (20–25°C). Finally pour the 100 μl of red stop solution into the wells, mix gently by sliding back and forth on a flat surface for 10–20 s without splashing reagents from the wells and read the optical density (OD) of standards and samples at 650 nm using a microplate reader.

**Figure 1:**
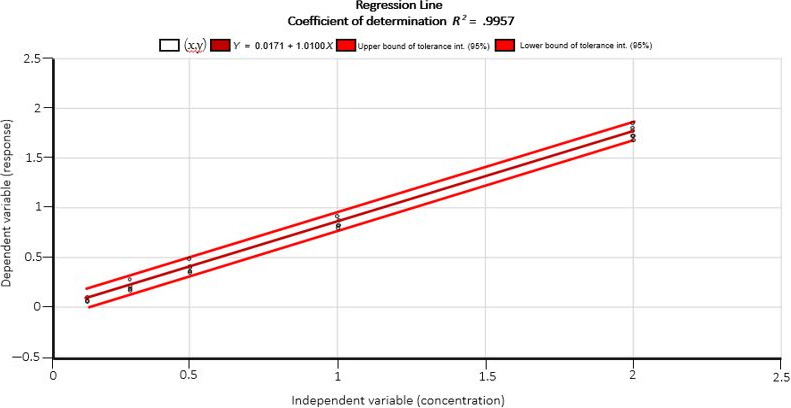
The plot of the regression line shows a linear response (*R* ^2^ > .991) within the working range studied (10–250 µg/kg)

A standard curve was constructed each time using series of standards of AFB1 solutions provided with the test kit (Table 2). The sample concentration was calculated based on the standard curve (Figure 1). The plot of the regression line showed a linear response (*r* ^2^ > .991) within the working range studied (10–250 µg/kg).

**Table 2:** Calibration data for coefficient of determination by linear model

Analyte	Unit	Concentration range		Slope		Intercept		Coefficient of determination *R* ^2^	Standard deviation of residuals
		Min	Max	Central value	Slope = 0? (Y/N)	Central value	Intercept = 0? (Y/N)		
AFB1	µg/L	0	50	1.02	N	0.41	N	0.991	2.006

In order to calculate precision, corn feed sample was analyzed as such and after spiking at three different AFB1 concentration levels (10, 50 and 250 µg/kg) on six different days, and each time in duplicate. Consequently, the number of AFB1 determinations at each level was 12, which gives 36 ELISA measurements for this matrix. We evaluated the percent coefficient of variation for repeatability (CVr) as 7.1%, 3.3% and 3.8% with percentage of coefficient variation as 14%, 3% & 8% while % recoveries were 101.25%, 99.22% and 104.0% for 10, 50 & 250 µg/kg spiked samples respectively (Table 3).

**Table 3:** Validation parameters of the ELISA method used for quantification of AFB1

Spiking levels (µg/kg)	No. of days * No. of replicates	Median	*SE*	Repeatability			CV (%)	Recovery (%)
				*SD*(r)	CV (r) (%)	R		
10	6*2	10.25	0.40	0.72	7.1	2.00	14	101.25
50	6*2	49.25	0.46	1.63	3.3	4.51	3	99.22
250	6*2	260	6.03	10	3.8	27.72	8	104.00

Abbreviations: *SD*, standard deviation, r, repeatability, *SE*, Standard Error, CV, coefficient of variation).

### 2.6 Statistical analysis

All data were presented as mean by using simple excel. Correlation was applied to calculate significant impact of temperature, precipitation, humidity on aflatoxin M1 in milk and & B1 in animal feed by SPSS, IBM PASW statistics 19, USA. Numerical variable data were presented in frequencies, percentages, and mean ± standard deviation. Bar chart were used to express total average and percentage of non‐compliant aflatoxin M1 (US MRL < 0.5 µg/L) and aflatoxin B1 (US MRL > 20 µg/kg) samples exceeding in selected clusters, while line charted indication levels of averages of aflatoxin M1 (µg/L) and aflatoxin B1(µg/kg) in different months of year 2015. Analysis of variance (ANOVA) was used for the comparison of average of aflatoxin M1 (µg/L) and aflatoxin B1 in different clusters. The results were considered statistically significant at *p* < .05. Correlation and regression analysis were applied to calculate R^2^ by using statistical package Q Stat.net.

## 3 RESULTS AND DISCUSSION

**Figure 2:**
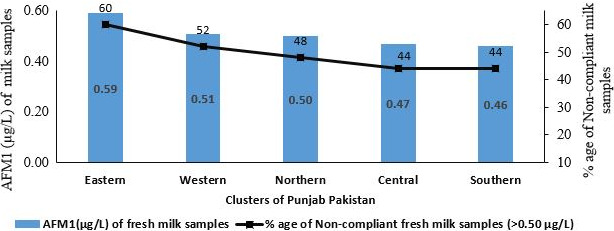
Percentage of raw milk samples exceeding US MRL (0.50 µg/L) for AFM1 & feed samples US MRL (20 µg/kg) for AFB1 the regulatory norms

**Figure 3:**
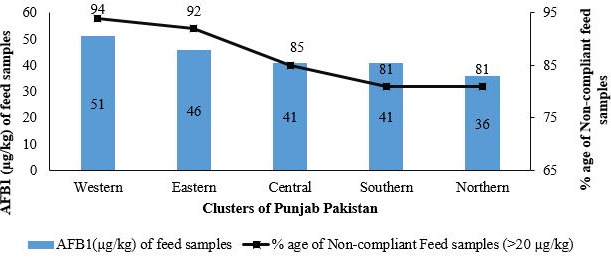
Season wise AFM1 (µg/L) in raw milk samples of farms among five clusters of Punjab Pakistan during year 2015 (*n* = 240 each)

In the present study it was observed that almost 53% of the milk samples collected from the dairy farms situated in the five clusters of Punjab province of Pakistan, were contaminated higher than safe limit of US < 0.5 µg/L (Figures 2 and 3). AFM1 contamination was observed highest in Eastern cluster (0.59 ± 0.03 µg/L) followed by Northern (0.51 ± 0.30 µg/L), Western (0.51 ± 0.30 µg/L) and Central (0.46 ± 0.30 µg/L) cluster, while Southern cluster (0.46 ± 0.30 µg/L) remained lower throughout the year as compare to the Eastern cluster (Table 4). Though the number of samples obtained from different clusters were same but availability of AFM1 was speckled. Average AFM1 contamination levels and number of samples exceeding US permissible limits were decreasing north to south as topography, temperature, rainfall, humidity and weather condition varies considerably. Despite of the fact that average AFM1 values of all clusters were statistically non‐significant. Current findings are more or less similar to those of Jawaid, Talpur, Nizamani, and Afridi (2015) where they reported 96.43% AFM1 contaminated samples with average contamination level of 0.38 µg/L. Our findings are consistent with studies from Greater Addis Ababa, where 26.3% milk samples collected from farms were found above the US permissible limits (Gizachew, Szonyi, Tegegne, Hanson, & Grace, 2016). Similar findings were reported by Iqbal and Asi (2013) where 71% milk samples were found contaminated (Rastogi, Dwivedi, Khanna, & Das, 2004). Aflatoxin contamination of raw, pasteurized and powdered milk samples from Syria market reported as 22%, 32% and 58% above the permissible limits of America, Syria and Europe respectively. Many of studies from Pakistan reported raw milk contamination as 58% (Iqbal & Asi, 2013), 72% for buffalos (Asi, Iqbal, Ariño, & Hussain, 2012), 53% (Ismail et al., 2016), 42% of milk samples from urban and 27% from rural (Iqbal & Asi, 2013) were well above the limit permitted by the European Union (EU). Study results from neighboring country India, reported 99% fresh milk samples exceeded Codex Limits (Rastogi et al., 2004).

**Table 4:** The occurrence of AFM1 in raw milk samples of dairy farms among five clusters of Punjab Pakistan during 2015 (*n* = 240)

Cluster	Total Samples	Months													Avg. AFM1 ± SD
			Jan	Feb	Mar	Apr	May	Jun	Jul	Aug	Sep	Oct	Nov	Dec	(µg/L)
Eastern	48	Avg. AFM1 (µg/L) (& No.of samples exceeding US MRL)	0.96 (4)	0.79 (4)	0.70 (4)	0.60 (3)	0.55 (3)	0.52 (3)	0.43 (2)	0.35 (0)	0.33 (0)s	0.43 (1)	0.59 (4)	0.82 (4)	0.59 ± 0.03 (32) AB
		Range (µg/L)	0.87–1.18	0.60–1.09	0.64–0.81	0.40–0.78	0.48–0.61	0.32–0.63	0.26–0.5	0.26–0.39	0.20–0.46	0.35–0.50	0.55–0.63	0.69–0.89	
Northern	48	Avg. AFM1 (µg/L) (& No.of samples exceeding US MRL)	0.69 (3)	0.66 (3)	0.63 (3)	0.55 (3)	0.53 (2)	0.45 (2)	0.38 (1)	0.35 (0)	0.27 (0)	0.35 (1)	0.53 (2)	0.68 (4)	0.51 ± 0.3C (24)
		Range (µg/L)	0.47–1.0	0.23–0.95	0.37–0.86	0.45–0.63	0.45–0.62	0.35–0.58	0.20–0.52	0.26–0.45	0.23–0.35	0.25–0.56	0.46–0.62	0.64–0.73	
Western	48	Avg. AFM1 (µg/L) (& No.of samples exceeding US MRL)	0.71 (4)	0.58 (4)	0.60 (3)	0.57 (4)	0.51 (2)	0.49 (2)	0.44 (1)	0.38 (0)	0.38 (1)	0.33 (0)	0.52 (2)	0.64 (3)	0.51 ± 0.02 A (26)
		Range (µg/L)	0.54–0.90	0.51–0.63	0.48–0.71	0.53–0.63	0.40–0.63	0.36–0.6	0.37–0.54	0.31–0.49	0.18–0.50	0.25–0.38	0.36–0.6	0.48–0.92	
Central	48	Avg. AFM1 (µg/L) (& No.of samples exceeding US MRL)	0.65 (3)	0.52 (3)	0.53 (3)	0.51 (3)	0.53 (3)	0.47 (2)	0.41 (0)	0.36 (0)	0.32 (0)	0.28 (0)	0.44 (2)	0.58 (4)	0.47 ± 0.02 BC (23)
		Range (µg/L)	0.47–0.80	0.37–0.63	0.38–0.60	0.41–0.56	0.45–0.62	0.38–0.60	0.38–0.44	0.34–0.38	0.18–0.48	0.22–0.36	0.29–0.61	0.50–0.66	
Southern	48	Avg. AFM1 (µg/L) (& No.of samples exceeding US MRL)	0.74 (4)	0.67 (3)	0.61 (4)	0.58 (3)	0.36 (0)	0.43 (1)	0.32 (0)	0.24 (0)	0.25 (0)	0.36 (0)	0.48 (3)	0.53 (3)	0.46 ± 0.03 BC (21)
		Range (µg/L)	0.52–0.96	0.48–0.85	0.51–0.73	0.31–0.84	0.25–0.47	0.25–0.64	0.17–0.43	0.14–0.35	0.15–0.37	0.29–0.43	0.26–0.60	0.32–0.64	
Overall	240	Avg. AFM1 (µg/L) (& No.of samples exceeding US MRL)	0.75 (18)	0.64 (17)	0.61 (17)	0.56 (16)	0.50 (10)	0.47 (10)	0.40 (4)	0.34 (0)	0.31 (1)	0.35 (2)	0.51 (13)	0.65 (18)	0.51 ± 0.03 (126)

Note

Means sharing similar letters are statistically non‐significant (*p* > .05).

Moreover, results showed that winter season is more critical entailing higher contamination of AFM1 and maximum number of samples exceeding permissible limits (Figures 2 and 3). Common months unveiling higher contamination in entire province regardless of division and clusters were December to March. Hence, severity of AFM1 in winter season collateral to Eastern cluster (Table 4). In current study, it is observed that farmer's offer contaminated stored and concentrate feed sources during winter due to the shortage of green fodder. Farmers in greed of more milk production during winter season increase the quantities of these available stored contaminated feed ingredients (i.e., corn, based, which ultimately increase the levels of AFM1 contamination (Asi et al., 2012). Therefore, quantities of feed are positively correlated to AFM1 (Table 6) in winter (*p* < .01). Feed consumed by animals is indeed the prime factor in this regard. Our study is supported by many previous studies of AFM1 contamination levels in winter season (Asi et al., 2012; Fallah, Rahnama, & Saei‐Dehkordi, 2011; Nemati, Mesgari Abbasi, Parsa Khankandi, & Masoud, 2010). Contrarily limited studies (Bahrami, Shahbazi, & Nikousefat, 2015; Fallah et al., 2011) reported no significant difference of season on AFM1 contamination levels.

**Figure 4:**
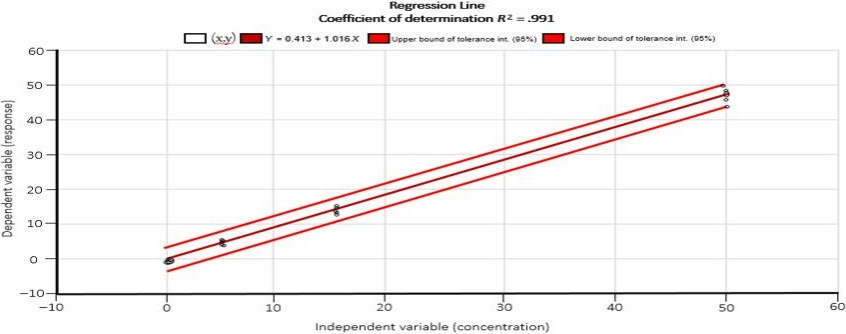
Occurrence of AFM1 (µg/L) in raw milk and AFB1 (µg/kg) of feed samples of farms among five clusters of Punjab Pakistan during year 2015 (*n* = 240 each)

It was extracted from current research that AFB1 concentration emerged in entire samples collected from five clusters, though concentration varied across the year. Monthly trend line of AFM1 and AFB1 levels showed that contamination of milk and feed are inter‐linked across the year (Figure 4). This concentration of AFB1 is solemnly associated with the feed consumed by the animals. Bread pieces, concentrate feed mix, maize and cotton seed cake were the often‐consumed feeds by the animals in all clusters. During informal discussion, it is observed that traditionally farmers assumed that use of cited feeds is helpful in elevating milk production and prefer to feed these one whenever available at cheaper rates.

In Western cluster about 94% feed samples were found AFB1 contaminated beyond US permissible limit (<20 µg/kg) with significantly (*p* < .05) higher average AFB1 levels (51 µg/kg) for feed. Eastern cluster exhibited 92% samples followed by 85%, 81% and 81% (Figure 2) contaminated samples exceeding allowable feed limits with average levels of 41, 41 and 36 µg/kg in Central, Southern and Northern clusters respectively. Average AFB1 contamination of all clusters were not significantly different from each other except Western cluster (Table 5).

**Table 5:** The occurrence of AFB1 in feed samples of dairy farms among five clusters of Punjab Pakistan during 2015 (*n* = 240)

Cluster	Total Samples	Months													Avg AFB1 ± SD
			Jan	Feb	Mar	Apr	May	Jun	Jul	Aug	Sep	Oct	Nov	Dec	(µg/kg)
Western	48	Avg. AFB1 (µg/L) (& No.of samples exceeding US MRL)	65.83 (4)	71.04 (4)	69.19 (4)	70.88 (4)	70.69 (4)	57.13 (3)	68.75 (4)	22.04 (3)	23.78 (2)	24.61 (4)	35.02 (4)	36.62 (4)	51 ± 3.82 A (45)
		Range (µg/L)	38–105	37–119	46–96	41–93	52–110	47–66	63–75	18–28	11–32	19–28	25–41	27–52	
East	48	Avg. AFB1 (µg/L) (& No.of samples exceeding US MRL)	44.60 (4)	51.77 (4)	57.96 (4)	59.33 (4)	68.65 (4)	53.48 (4)	57.80 (3)	20.01 (1)	20.48 (1)	31.64 (4)	40.29 (3)	46.74 (3)	46 ± 2.75 B (44)
		Range (µg/L)	36–57	42–63	46–76	50–79	62–82	18–76	23–79	15–23	13–29	26–37	37–43	39–51	
Central	48	Avg. AFB1 (µg/L) (& No.of samples exceeding US MRL)	73.83 (4)	37.17 (4)	34.78 (4)	41.90 (4)	54.81 (4)	62.80 (4)	65.08 (4)	21.03 (3)	20.00 (3)	20.54 (3)	30.09 (4)	33.20 (4)	41 ± 2.98 B (41)
		Range (µg/L)	53–94	22–48	25–39	29–61	43–77	52–77	45–82	20–23	11–30	16–27	20–42	29–38	
Southern	48	Avg. AFB1 (µg/L) (& No.of samples exceeding US MRL)	34.91 (4)	49.43 (4)	52.54 (4)	62.60 (4)	58.71 (4)	62.34 (4)	47.25 (4)	13.78 (3)	15.75 (1)	26.64 (2)	32.81 (2)	32.92 (4)	40 ± 2.87 B (39)
		Range (µg/L)	25–44	44–56	48–56	52–77	34–76	36–74	19–73	8–20	10–24	22–32	18–42	18–47	
Northern	48	Avg. AFB1 (µg/L) (& No.of samples exceeding US MRL)	34.23 (4)	43.45 (4)	47.64 (4)	48.00 (4)	53.48 (4)	30.26 (3)	46.84 (4)	17.96 (1)	17.01 (1)	25.90 (2)	32.39 (2)	36.64 (4)	36 ± 2.35 B (39)
		Range (µg/L)	34–53	26–63	26–66	32–76	37–68	15–53	32–63	15–20	15–22	19–41	21–42	32–41	
Overall	240	Avg. AFB1 (µg/L) (& No.of samples exceeding US MRL)	50.68 (20)	50.57 (20)	52.42 (4)	56.54 (20)	61.27 (20)	53.20 (18)	57.14 (19)	18.96 (11)	19.40 (19)	25.86 (26)	34.12 (2)	37.22 (19)	43 ± 2.87 (228)

Note

Means sharing similar letters are statistically non‐significant (*p* > .05).

Levels of AFB1 of Western cluster was more critical having maximum average AFB1 71.04 µg/L (range 36.54–119 µg/L) in the month of February followed by the month of May 0.70.69 µg/kg (range 51.66–110.2 µg/kg) while Northern cluster has lowest contaminated feed samples with highest AFB1 48 µg/kg (range 31.5–76.4 µg/kg) in the month of April followed by March AFB1 47.64 µg/kg (range 26–66.4 µg/kg). Result of all clusters (Table 5) indicated that maximum contaminated feed samples were observed during the months of January to July, when most of the time stored feed ingredients consumed in the form of concentrate. In Pakistan, key feed ingredients directly or the part of concentrate feed are maize and cottons seed cake of which crops harvested during the month of August to September. During this time, hot and humid weather prevailing with rainfall contaminate these feed sources. These feed ingredients are stored and used in later months. These finding are aligned with Kamkar, Karim, Aliabadi, and Khaksar (2008), who observed increase in the AFB1 contamination with delayed storage and high moisture. Many studies supported the direct relationship between AFB1 contamination and storage time (Rastogi et al., 2004). Smith and Moss (1985) reported the impact of temperature, humidity, handling and harvesting conditions on high levels of aflatoxin contamination during storage of feed. The storage impact on AFB1 has been demonstrated by many other studies (Azziz‐Baumgartner et al., 2005; Mwalwayo & Thole, 2016).

Maize is preferred feed for farmers at dairy farms. Maize samples used for animals feed also appeared source of aflatoxin (Ahsan, Bhatti, Asi, Bhatti, & Sheikh, 2010). Maize crop through fungi could develop aflatoxin concentration of varied level (Sanchis & Magan, 2004). Several researches (Anjum, Khan, Sahota, & Sardar, 2012; Bhatti, Talat, & Sardar, 2001) found highest contamination of aflatoxin in corn, which is common feedstuff for animals. Reddy and Salleh (2011) reported 23% AFB1 contaminated samples ranging from 21 to 135 µg/kg. Similar findings were reported 61% maize samples AFB1 contamination above the permissible limits (Anjum et al., 2012). Farmers also offer bread pieces to animals, which are waste material and high source of aflatoxin contamination. Asi et al. (2012) reported that those animals fed on bread pieces and concentrates, evolve higher amount of aflatoxin in milk. Chauhan, Washe, and Minota (2016) also revealed that dairy concentrates were highly contaminated (64%) feedstuff for animals.

A significant association (*p* < .05) between precipitation and emergence of aflatoxin in milk and feed during winter, spring and summer season was observed (Table 6). However, during autumn season precipitation did not exhibited any influence on aflatoxin in milk and feed. In overall context, association appeared significant. Temperature indicated significant but negative association with aflatoxin in milk during autumn season. Lower temperature promotes fungal growth and AFB1, hence, findings do not corroborate with those of Pratiwi et al. (2015). Humidity, another determinant of environment was significantly associated (*p* < .05) with aflatoxin generation in feed during winter, spring and summer season. Findings regarding humidity are in alliance with of Pratiwi et al. (2015) where they unveiled AFB1 incidences pertinent to humidity. In Pakistan feed contamination variation were attributed to persistent relative humidity and rainy season (especially hot monsoon) which usually persist between June to September (Anjum et al., 2012; Rashid et al., 2012; Yunus, Nasir, Aziz, & Böhm, 2009). The season has a significant role on fungal growth. In the current study data (Figure 3) depicts that rainy season (Jun to Sep) effected the crops especially corn & cotton. Later on animals feed on these sources were significantly raised the levels of AFB1 & AFM1 in upcoming months of winter (Figure 3). The AFB1 contamination levels are influenced by environmental conditions like humidity and temperature (Hanif, 2009; Hanif et al., 2008). Similarly in another study it is reported that the corn harvested during wet season had higher (66.4 µg/kg) AFB1 level than harvested in dry season (37 µg/kg; Tangendjaja, Rachmawati, & Wina, 2008). Results (Table 6) are evident that there is significant impact of environment on aflatoxin concentration. These findings are contradicting to those of Chauhan et al. (2016) where they reported high level of AFB1 in the months from June to November while low concentrations were perceived during December to May.

**Table 6:** Correlation between seasonal variations and different factors (Environmental & physical) effecting aflatoxin levels in Punjab Pakistan during 2015

Factors	Winter		Spring		Summer		Autumn		Overall	
	M1	B1	M1	B1	M1	B1	M1	B1	M1	B1
Environmental factors										
Precipitation	0.164*	0.049**	0.300*	0.222**	0.401*	0.097*	0.395	0.051	0.402**	0.011**
Temperature	−0.424	0.155	−0.372	−0.176	0.337	−0.234	−0.889**	0.190	−0.693*	−0.089
Humidity	0.119	0.304*	0.216	0.175*	−0.589**	−0.273*	−0.052	0.432	0.202*	0.258*
Physical factors										
Feed quantity (BP)	–	–	–	–	0.274	0.813*	–	–	0.245	−0.437
Feed quantity (CFM)	0.311	0.646	0.786*	0.764*	0.274	0.813*			0.118	0.044
Feed quantity (Corn)					−0.419	−0.434			−0.420	−0.054
Feed quantity (CSC)	0.496*	0.015**	0.188	0.417	0.708**	0.323*	0.262	−0.034	0.374**	0.027**
Feed quantity (Average)	0.441	0.248**	0.184	0.780**	0.647**	0.338*	0.346	0.054	0.394**	0.020*
Feed price (BP)					0.897*	0.270			0.848*	0.179
Feed price (CFM)	0.664	−0.541	0.370	−0.340	−0.258	−0.351			0.080	−0.341
Feed price (Corn)					0.074	0.276			0.098	0.010*
Feed price (CSC)	0.133	0.408*	0.179	0.087	0.724**	0.253	0.275	−0.015	0.407**	0.015*
Feed price (Average)	0.619*	−0.273*	0.342	−0.372	−0.591**	−0.362*	0.323	0.048	0.441**	−0.168*

* Significant (*p* < .05).

** Highly significant (*p* < .01).

In current study, quantity of concentrated feed appeared significantly associated with aflatoxin development in animals feed and milk simultaneously during spring season. In Punjab province concentrate remained a preferred feed when the green fodder shortage observed. These concentrates are more susceptible to fungal growth which are in line with the studies of Asi et al. (2012) who reported the high levels of AFM1 in milk of animals fed on these concentrate. Similarly it was observed that cotton seed cake remained preferred animal feed throughout the year. Overall quantity of cotton seed cake was positively associated with AFM1 (*p* < .01) and AFB1(*p* < .05) contamination levels. Traditionally this considered as high‐energy source among small farmers for animal feed particularly after calving stage. These findings are similar to those of Ullah et al. (2016) where highest aflatoxin concentration was found in cotton seed cake as compared to *wanda* or concentrate feed mixtures. Findings of the Anwari, Ullah, and All (2013) concluded that consumption of cotton seed cake increased milk production and fat concentration in milk. Cotton seed cake is pleasant source of protein and encouraging milk production. Cotton seed cake served as appetizer for animals (Yasmeen et al., 2007). Positive impact of cotton seed cake on weight of lactating animals has been proven by number of research studies as well (Chowdhury, 2001; Jabbar, Anjum, Rehman, & Shahzad, 2006; Jabbar & Marghazani, 2009). Despite of extensive significance in health improvement and milk production, cotton seed cake produce AFM1 in milk of animals. Ullah et al. (2016) identified highest concentration of AFB1 in lactating animals fed on cotton seed cake. Cotton seed cake happened to be a source of fungi because of excessive proteins and lipids inside. Chauhan et al. (2016) found highest contamination (68%) of aflatoxin in cotton seed cake. Saleemi, Khan, Khan, and Javed (2010) reported that cotton seed cake is most susceptible to fungal attack, which is prime source of aflatoxin.

**Figure 5:**
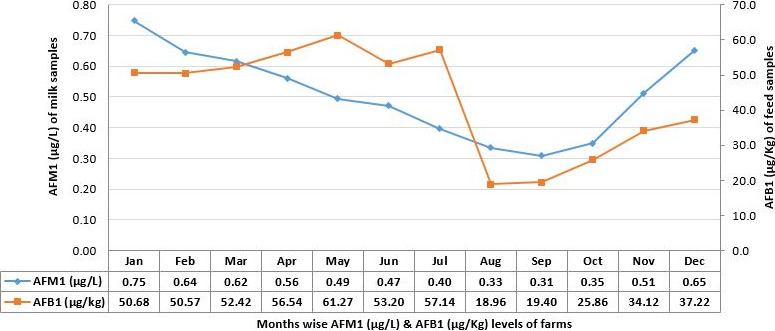
Correlation of feed quantity and feed price procured at farm during all seasons of five clusters of Punjab Pakistan during 2015

In addition to above details, the average prices of feed sources had negative correlation with AFB1 contamination levels (Figure 5) which were further supported by the positive correlation between quantities of feed at farms. This indicated that in case of high prices, farmers opt an alternative cheaper source of feed regardless of aflatoxin contamination.

## 4 CONCLUSION

This comprehensive investigation examined the occurrence of aflatoxins contamination parallel in the milk and animal feed of dairy farms of Punjab province of Pakistan covered all season during the complete year 2015. It is observed that majority of samples collected from five clusters of Punjab were contaminated with aflatoxin above the US permissible limits. Local regulations for AFB1 levels of feed ingredients are dire need to revise as with current levels, milk of desired AFM1 levels (<0.50 µg/L) could not be delivered. Further regular monitoring of not only AFM1 in milk, but also the AFB1 in feed shall need to strengthen. Impact of environmental factors on concentration of aflatoxins were significant. Seasonal variations unveiled that precipitation indicated highly significant impact on aflatoxin concentration in milk and feed which directing authorities and policy makers attention to facilitate farmers towards comprehensive post‐ harvest management programs. These strategies will helpful to lower down the toxins burden in feed and raw milk supply chain. Future studies could possibly focus aflatoxin contamination contribution levels of all feed sources including silage, fodder and other feed ingredients which might use at farms.

## CONFLICT OF INTEREST

Authors declare that they have no conflict of interest.

## ETHICAL APPROVAL

This article does not contain any studies with human participants or animals performed by any of the authors.

## INFORMED CONSENT

For this type of study, formal consent is not required.
